# Spleen injury following left extracorporeal shockwave lithotripsy (ESWL)

**DOI:** 10.1186/1471-2490-15-4

**Published:** 2015-02-18

**Authors:** Serge P Marinkovic, Christina M Marinkovic, Donghua Xie

**Affiliations:** Department of Urology, Detroit Medical Center-Harper and Hutzel Hospitals, Detroit, Michigan 48201 USA

**Keywords:** Splenic injury, Splenectomy, ESWL

## Abstract

**Background:**

A splenic rupture associated with extracorporeal shockwave lithotripsy (ESWL) is exceedingly rare. We report a case of stage 3 splenic laceration, hemoperitoneum and subsequent splenic rupture following an ESWL for a left mid polar renal calculus.

**Case presentation:**

During the ESWL, although the patient’s pain was controlled the gentleman was very nervous and had to be repositioned eight individual times.

Approximately 6 hours after the ESWL, the patient phoned the urologist complaining of severe left flank pain unlike any previous episode of renal colic. A computerized tomography (CT) scan demonstrated a stage 3 splenic injury with hemoperitoneum. The patient decompensated and an emergent splenectomy was then performed and the patient experienced an uneventful recovery.

**Conclusions:**

Splenic injury likely results from unintentional movement during the sound wave administration for the stone fragmentation procedure. Utilizing noise cancelling headphones during ESWL may preclude the potential pitfalls of patient nervousness.

## Background

A splenic rupture associated with extracorporeal shockwave lithotripsy (ESWL) is exceedingly rare [1–6]. The literature does not report comprehensive preponderance of evidence for this occurrence unless related to portal hypertension with severe coagulopathy [5].

## Case presentation

**Figure 1:**
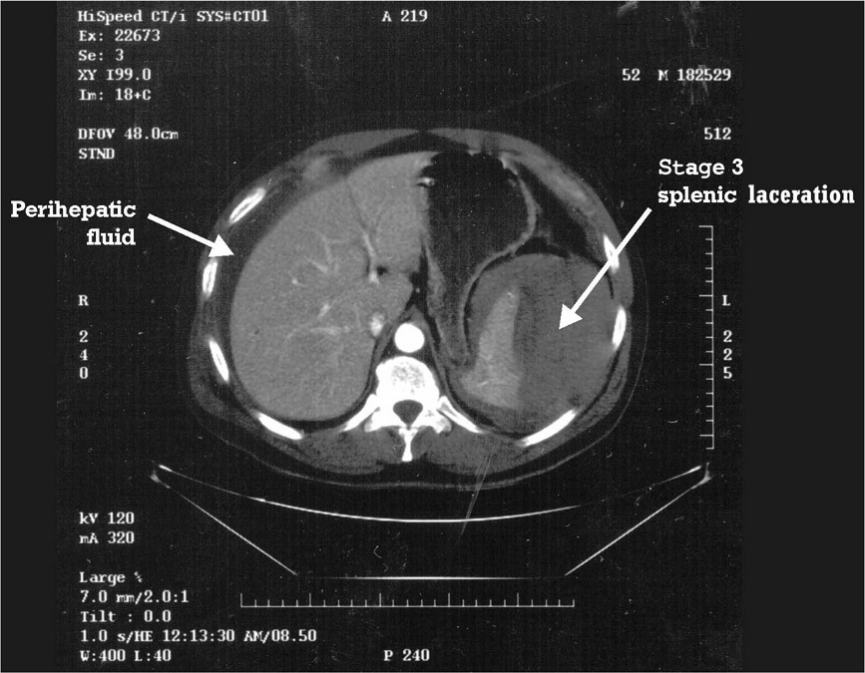
**CT scan: stage 3 splenic injury with hemoperitoneum.**

**Figure 2:**
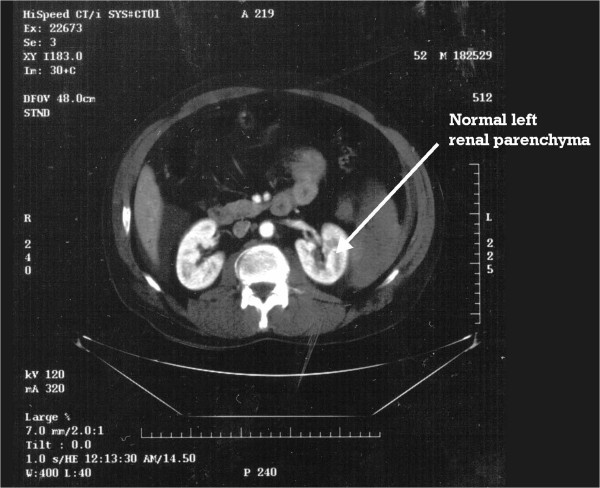
**CT scan: the left kidney noted no extravasation or parenchymal injury but with a fractured calculus.**

A 54-year-old anxious male presented to the emergency room after having 12 hours of severe medically recalcitrant left flank pain. A CT scan and KUB demonstrated a 15 mm by 8 mm left mid polar renal calculus and the patient underwent left ESWL under regional anesthetic, which was performed with 1–7 KV power, at 120 shocks per minute for a total of 2500 shocks. Although the patient’s pain was controlled the gentleman was very nervous and had to be repositioned eight individual times.Approximately 6 hours after the ESWL, the patient phoned the urologist complaining of severe left flank pain unlike any previous episode of renal colic. An emergency room evaluation noted a hypotensive patient with left upper and lower quadrant pain and peritoneal signs. A CT Scan demonstrated a stage 3 splenic injury with hemoperitoneum (Figure 1) while the left kidney noted no extravasation or parenchymal injury but with a fractured calculus illustrating that the stone was effectively treated (Figure 2). A general surgery consultation was obtained, and the general surgeon performed an emergent splenectomy and drained 2.0 liters of hemoperitoneum while transfusing the patient with six units of packed red blood cells. The patient experienced an uneventful recovery.

## Discussion

Although ESWL is a relatively safe method in the treatment of urinary stones, rare complications like splenic rupture could occur. Special attention should be given to patients with kidney stones in the left upper or middle pole, pathological growth or unusual position of the spleen, accompanying disease such as portal hypertension with severe coagulopathy [2, 4, 5]. Our patient felt no pain under his regional anesthetic although he anxiously responded with side-to-side movement to the loud, tapping sound of the Dornier Compact Delta lithotriptor. Even with frequent repositioning of the patient, unexpected direct or reflected acoustic sound waves may have injured the posterior side of the spleen at the retroperitoneal reflection of the left kidney. We have performed over 600 ESWLs utilizing noise cancelling headphones to preclude the potential pitfalls of loud acoustic sound waves causing patient nervousness during ESWL.

## Conclusions

Splenic injury likely results from unintentional movement during the sound wave administration for the stone fragmentation procedure. Utilizing noise cancelling headphones during ESWL may preclude the potential pitfalls of patient nervousness.

## Consent

Written informed consent was obtained from the patient for the publication of this report and any accompanying images.

## Abbreviations

ESWL: Extracorporeal shockwave lithotripsy
CT: Computerized tomography
KUB: Kidneys, ureters, and bladder x-ray.
